# Association of combination cardiovascular therapies with cognitive decline and neuropathologies

**DOI:** 10.1002/alz70860_106195

**Published:** 2025-12-23

**Authors:** Roshni Biswas, Ana W. Capuano, Rupal I. Mehta, David A. A. Bennett, Zoe Arvanitakis

**Affiliations:** ^1^ Rush Alzheimer's Disease Center, Rush University Medical Center, Chicago, IL, USA; ^2^ Rush University Medical Center, Chicago, IL, USA

## Abstract

**Background:**

Hypertension, dyslipidemia, and diabetes are known cardiovascular risk factors for dementia. Anti‐hypertensive, lipid‐lowering, and anti‐diabetes medications have been proposed as potential therapies to mitigate dementia risk. Here, we assess the impact of combination therapies across these three medication classes on cognitive decline and dementia‐related postmortem neuropathologies.

**Method:**

Participants included 4651 older adults from five cohort studies of aging with no baseline dementia and at least two annually‐collected global cognition measures. Visually‐inspected medications were documented annually. The indicator for combination therapies with anti‐hypertensive, lipid‐lowering, and anti‐diabetes medications was the sum of the number of therapies by class (range:0‐3). Mixed effects models evaluated associations of baseline therapies with cognitive decline. On a subgroup of 1896 deceased and autopsied participants, neuropathologic evaluations documented cerebrovascular disease, Alzheimer's disease (AD), and other dementia‐related neuropathologies. Linear and logistic regression models evaluated associations of therapies at the last clinical evaluation proximate‐to‐death with neuropathologies. All models adjusted for age, sex, and education.

**Result:**

Compared to no medication use, baseline therapies with three medication classes were associated with slower decline in global cognition (estimate=0.02;p=0.02), particularly semantic and working memory. Among autopsied participants, therapies with three medication classes were associated with lower odds of atherosclerosis (OR=0.47;p<0.01) and arteriolosclerosis (OR=0.53;p=0.01), but higher odds of brain infarcts (OR=1.74;p=0.01), particularly macroinfarcts (OR=1.66;p=0.03), as well as with less global AD pathology (estimate=‐0.22; *p* <0.01), specifically amyloid and tangles, lower odds of TDP‐43 (OR=0.46;p<0.01) and hippocampal sclerosis (OR=0.27;p=0.03). Baseline therapies with two medication classes were associated with slower decline in global cognition (estimate=0.01;p< 0.01), particularly episodic, semantic and working memory. Among autopsied participants, therapies with two medication classes were associated with lower odds of atherosclerosis (OR=0.63;p<0.01), less global AD pathology (estimate=‐0.09;p=0.03) and tangles (estimate=‐0.22; *p* = 0.03), and lower odds of TDP‐43 (OR=0.71;p=0.02). Baseline therapy with one medication class was associated with slower semantic memory decline (estimate=0.10; *p* = 0.02) and in autopsied participants, with less tangles (estimate=‐0.20;p=0.02) and lower odds of TDP‐43 (OR=0.73;p=0.02).

**Conclusion:**

Combination therapies with anti‐hypertensive, lipid‐lowering, and anti‐diabetes medications are associated with slower cognitive decline and less dementia‐related neuropathologies. Combination therapies targeting vascular metabolic risk factors have the potential to prevent dementia.

